# Pressure drop of two-phase helium along long cryogenic flexible transfer lines to support a superconducting RF operation at its cryogenic test stand

**DOI:** 10.1186/s40064-016-3717-9

**Published:** 2016-12-05

**Authors:** M. H. Chang, M. H. Tsai, Ch. Wang, M. C. Lin, F. T. Chung, M. S. Yeh, L. H. Chang, C. H. Lo, T. C. Yu, L. J. Chen, Z. K. Liu

**Affiliations:** National Synchrotron Radiation Research Center, 101, Hsin-Ann Road, Hsinchu Science Park, Hsinchu, 30076 Taiwan, ROC

**Keywords:** Pressure drop, Cryogenic transfer line, Helium, SRF module, Two-phase flow

## Abstract

**Background:**

Establishing a stand-alone cryogenic test stand is of vital importance to ensure the highly reliable and available operation of superconducting radio-frequency module in a synchrotron light source. Operating a cryogenic test stand relies strongly on a capability to deliver two-phase helium along long cryogenic transfer lines. A newly constructed cryogenic test stand with flexible cryogenic transfer lines of length 220 m at National Synchrotron Radiation Research Center is required to support a superconducting radio-frequency module operated at 126.0 kPa with a 40-W dynamic load for a long-term reliability test over weeks. It is designed based on a simple analytical approach with the introduction of a so-called tolerance factor that serves to estimate the pressure drops in transferring a two-phase helium flow with a substantial transfer cryogenic heat load. Tolerance factor 1.5 is adopted based on safety factor 1.5 commonly applied in cryogenic designs to estimate the total mass flow rate of liquid helium demanded. A maximum 60-W dynamic load is verified with experiment measured with heater power 60 W instead after the cryogenic test stand has been installed.

**Results:**

Aligning the modeled cryogenic accumulated static heat load with the results measured in situ, actual tolerance factor 1.287 is obtained. The feasibility and validity of our simple analytical approach with actual tolerance factor 1.287 have been scrutinized by using five test cases with varied operating conditions. Calculated results show the discrepancies of the pressure drops between the estimated and measured values for both liquid helium and cold gaseous helium transfer lines have an underestimate 0.11 kPa and an overestimate 0.09 kPa, respectively. A discrepancy is foreseen, but remains acceptable for engineering applications from a practical point of view.

**Conclusions:**

The simple analytical approach with the introduction of a tolerance factor can provide not only insight into optimizing the choice of each lossy cryogenic piping element of the transfer lines in the design phase but also firm guidance for upgrading the present cryogenic transfer lines for its subsequent application.

## Background

Since its first introduction in Taiwan Light Source (TLS) at National Synchrotron Radiation Research Center (NSRRC) (Jensen 2011), 500-MHz superconducting radio-frequency (SRF) modules are currently widely selected as RF accelerating cavities for newly constructed or upgraded third-generation synchrotron light sources including Canadian Light Source (CLS, Canada), SOLEIL synchrotron (SOLEIL, France), Diamond Light Source (DLS, UK), Shanghai Synchrotron Radiation Facility (SSRF, China), Pohang Light Source II (PLS-II, Korea), National Synchrotron Light Source II (NSLS-II, USA) and Taiwan Photon Source (TPS, Taiwan). These SRF modules are all operated with dedicated closed-loop 4.5-K helium cryogenic plants. To fulfill the strict requirements of operational reliability and availability for users of synchrotron light sources, continuously improving or upgrading the operational performance of a SRF module has been considered necessary. Considering also the limited operational life expectancy of a SRF module, developing a stand-alone cryogenic test stand in house is commonly necessary to verify both the performance and the expected improvements, or a reliable examination of a SRF module that might be either newly constructed or repaired or to serve as a spare.

The cryogenic plant placed in service for the SRF module(s) on duty used in a synchrotron light source is typically oversized, following a standard rule of thumb commonly adopted to select a cryogenic plant with safety factor 1.5 for its refrigeration capacity. The inclusion of this safety factor opens a possibility to support a cryogenic test stand in a parasitic mode while maintaining an uninterrupted operation of the SRF modules on duty. The location of the cryogenic plant is commonly selected to be as near the SRF modules on duty as practical for the light source to benefit the routine operational efficiency, but the cryogenic test stand might be compelled to be located some distance from the main Dewar (MD) of the cryogenic plant because of space limitations. Under these circumstances, it becomes unavoidable to deliver liquid helium (LHe) from MD of a cryogenic plant to the tested SRF module at the cryogenic test stand to maintain the level balance of the SRF module and to send the evaporated cold gaseous helium (CGHe) from the tested SRF module to the cold box (CB) of a cryogenic plant to maintain the pressure balance of a SRF module over a long distance. Some operational challenges are consequently expected, such as insufficient pressure drops and a deterioration of the refrigeration capacity of the cryogenic plant. Both situations are induced mainly by the unexpected heat loads in the LHe and CGHe transfer lines. Such unexpected heat loads are easily introduced into a long-distance cryogenic transfer line. It might be questioned whether the guideline with safety factor 1.5 to account for the uncertainty of the heat load in the cryogenic designs is applicable also to the design of a long-distance cryogenic transfer line. Its validity must be verified for a design concept of this kind applied to long-distance cryogenic transfer lines of two-phase helium flow.

The maximum deliverable rate of flow of LHe reserved for the operation of the tested SRF modules relies strongly on the available budget of the pressure drop. The acceptable operating pressure of the tested SRF module places a constraint on the pressure drops of the cryogenic transfer lines. As these lines suffer from unexpected pressure drops resulting from a transfer heat load or other causes, the maximum deliverable rate of helium flow falls short. An unaccountable heat load is easily introduced through various constraints at the cryogenic piping interfaces, an inappropriate piping path from constraints in civil engineering, assembly defects from human negligence etc. This extra heat load might transcend the original expectation to neglect the existence of a two-phase fluid during the transfer of LHe and oversimplify the calculation of the pressure drop with a single-phase flow model. A two-phase flow model must thus be considered in evaluating the pressure drop that becomes much larger than that obtained with a single-phase flow model. If the pressure drops of LHe and CGHe transfer lines are underestimated with a large error, the maximum accelerating gradient of the tested SRF module to be examined at the cryogenic test stand becomes significantly compromised because of either an unacceptable over-pressure operation or an insufficient cooling capacity. In the worst case, a test of the long-term reliability of the tested SRF module becomes prohibitive because of a conflict with the cryogenic operation of the SRF modules on duty in the synchrotron light source. To avoid the above-mentioned problems, we must ensure that the pressure drops of long-distance cryogenic transfer lines are designed to assure a total mass flow rate of LHe to meet the requirement of the cryogenic test stand. How to design cryogenic transfer lines over a long distance is an important challenge in establishing a cryogenic test stand. Three important factors must be taken into consideration: first, the maximum allowable pressure drops of cryogenic transfer lines must be specified depending on the operating pressure of the tested SRF module; second, plausible values of heat loads in the LHe supply lines must be estimated because they dominate the magnitude of the total mass flow rate; third, a two-phase flow model to calculate the pressure drops of long cryogenic transfer lines with acceptable uncertainty must be available or developed.

The complication of calculating the pressure drop of a transfer line arises from the dynamic behavior of a two-phase helium flow along the cryogenic transfer line. There exist two available theoretical models for a calculation of the pressure drop of two-phase flow in a pipe, namely the classical Martinelli–Nelson equation (Martinelli and Nelson 1948) and the homogeneous equilibrium model (Collier 1981). The pressure drop of a turbulent two-phase helium flow in a horizontal channel has been addressed with these two methods; experiments have been conducted to verify the same (Vishnev et al. 1982). Another relevant paper by Rane et al. (2011) reports a successful use of numerical simulation to modify the classical Martinelli–Nelson equation, and demonstrates the equivalence between the classical Martinelli–Nelson equation and the homogeneous equilibrium model. It also reproduces the pressure drop in helium transfer lines in experimental tests by Vishnev et al. (1982) and Mamedov et al. (1983). With the rapid development of computational speed in a computer, a homogenous two-phase dynamic model based on continuity of mass, momentum and energy with pressure–volume-temperature relations (Regiera et al. 2011) is directly solvable after numerical discretization, which provides a theoretically reliable and accurate approach but at the cost of heavy computational resources. We prefer to apply a reliable, and approximate but efficient, approach involving only algebraic formulae to predict the pressure drop to minimize the design cost. Our theoretical model to calculate the frictional pressure drop of two-phase flow adopts a homogeneous equilibrium model with a new definition of Reynolds number and the frictional factor of two-phase flow, which was proposed by Shannak (2008), as presented in “Theoretical model for varied pressure drops” section below.

One must still determine the total heat load, i.e. the total mass flow rate, demanded under the test of a SRF module, which is used in the calculation of pressure drops of long-distance cryogenic transfer lines. The uncertainty of an estimate of the total heat load arises mainly from the heat loads of the LHe transfer lines. We aligned the accumulated static heat load from the engineering specifications of each LHe supply line provided by vendors with the measured value of a cryogenic transfer system as built. A tolerance factor is introduced in our alignment effort to take into consideration all construction uncertainties of the heat load and is also an effective merit of figure to verify the applicability of our calculational approach. Tolerance factor 1.5 is adopted as the safety margin of heat load of a LHe supply line to determine the total mass flow rate demanded in the test of a SRF module in the design phase, as is commonly used in cryogenic designs. After the transfer system is installed, the value of the actual tolerance factor can be verified on aligning the modeled accumulated static heat load with the results measured in situ. Additionally, after the actual accumulated static heat load from the LHe supply line is obtained, the actual total heat load at varied heater power applied on the tested SRF module will be deduced. In this paper, we take the 220-m flexible cryogenic transfer lines as built at NSRRC as an example to introduce how to design long cryogenic transfer lines for a cryogenic test stand. This transfer system is originally designed with tolerance factor 1.5; the actual tolerance factor is found to be 1.287 by experiment after the system installation. The feasibility and validity of our simple analytical approach with actual tolerance factor 1.287 are verified using five test cases with varied operating conditions.

The structure of this paper is as follows. “Cryogenic test stand and its configurations” section describes the cryogenic layout to be discussed in this work. “Design process and test results of the cryogenic test stand” section explains the design process and the test results of the cryogenic test stand at NSRRC. “Results of experimental measurements” section summarizes the measured results from the cryogenic transfer lines as built. “Theoretical model for varied pressure drops” section presents our computational approach to determine algebraically the pressure drops of the two-phase helium flow along a LHe supply line. “Heat loads of the cryogenic transfer lines” section introduces the concept of a tolerance factor and describes how to align it with the measured results. We discuss the results in “Results and discussion” section before a conclusion in “Conclusions” section.

## Cryogenic test stand and its configurations

NSRRC operates two 470-W, 4.5-K helium cryogenic plants (Hsiao et al. 2008) simultaneously for the electron storage ring of TLS. During the standard operational scenario, CB #1 is to support the routine operation of the SRF module on duty (named S1) and CB #2 for five superconducting magnets. The latter serves also as a backup cryogenic plant for the SRF module on duty. This redundant design ensures a highly reliable and uninterrupted cryogenic operation of the SRF module and for the light source. The unloaded refrigeration capacity was originally designed to be sufficient to support one additional tested SRF module (named S0) operated at a large accelerating gradient for a long-term test run at its cryogenic test stand.

Rigid multi-channel lines are commonly selected to transfer LHe over a long distance to take advantage of a small pressure drop and transfer heat load, but the piping path from the TLS cryogenic plant to the cryogenic test stand must pass through three separate buildings with varied piping elevation over a total piping length greater than 220 m. LN_2_-shielded, flexible cryogenic transfer lines of corrugated type were eventually chosen as a compromise considering several factors including the availability of the piping path, and quick, easy and cheap installation (Laeger et al. 1978; Blessing et al. 1990).

**Fig. 1:**
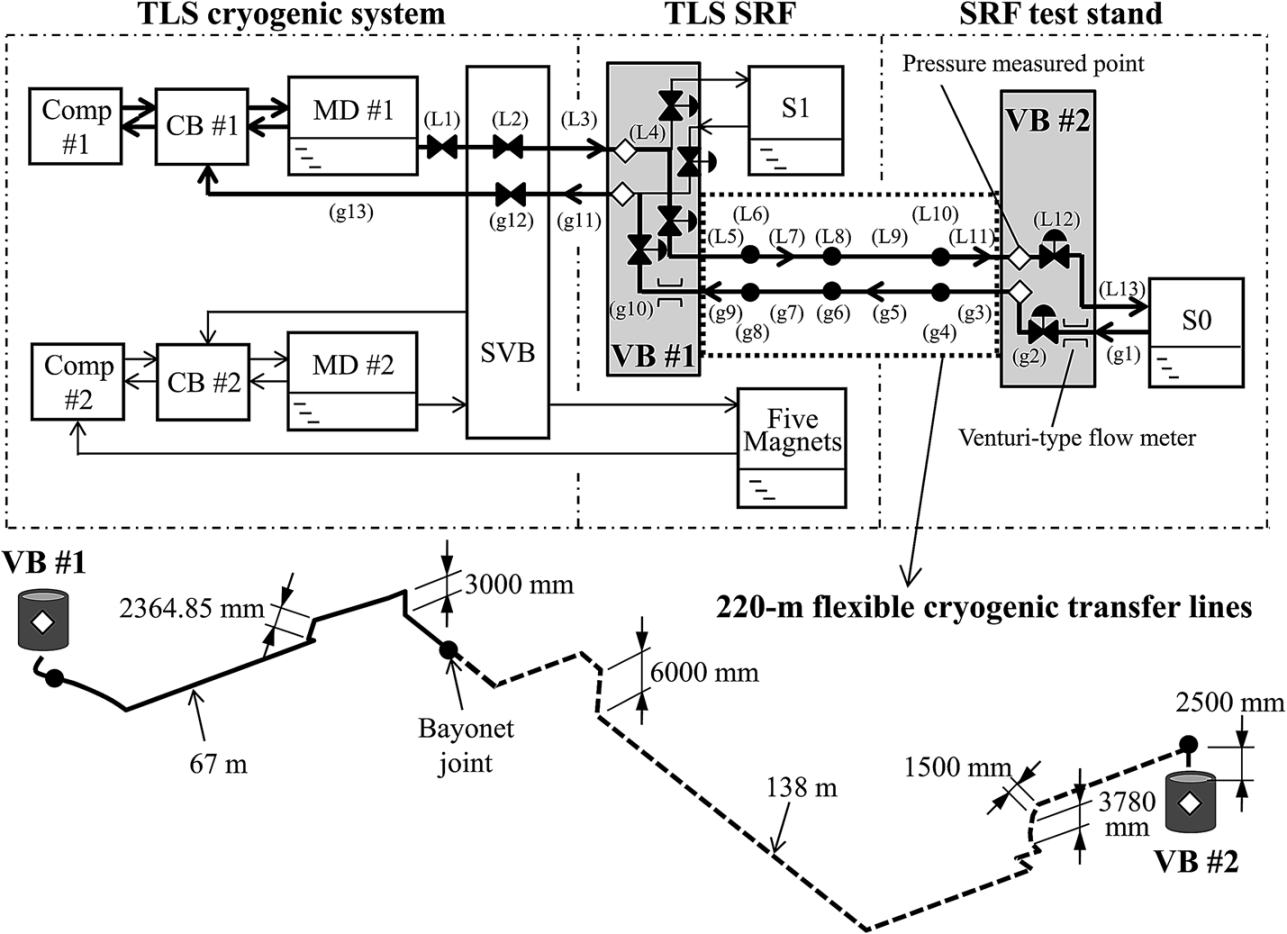
Simplified schematic diagram of the cryogenic plants with cryogenic transfer lines and piping path of flexible cryogenic transfer lines of length 220 m to operate the tested SRF module S0 at NSRRC

Figure 1 illustrates the simplified schematic diagram of the cryogenic plants with the cryogenic transfer lines to operate the SRF module under test at the cryogenic test stand. The required LHe for S0 is delivered from MD #1 of CB #1 to the LHe vessel of S0 through the LHe supply lines from L1 to L13; the evaporated CGHe is returned to CB #1 through the CGHe return lines from g1 to g13. For simplicity of illustration, the cryogenic valves directly after MD #2 and the detailed piping inside the switching valve box (SVB) are omitted from the details in Fig. 1, because their pressure drops are irrelevant for our discussion.

Cryogenic transfer lines L1 to L4, L12, g2, and g10 to g13, are solid pipes with smooth surfaces, but L5 to L11 and g3 to g9, between the first valve box, VB #1, and the second valve box, VB #2, are all corrugated flexible transfer lines. Lines L7 and L9, as well as g5 and g7, are concentric lines of four-tube design (CRYOFLEX) (Laeger et al. 1978; Blessing et al. 1990) with integrated length 205 m, whereas L6, L8, L10, g4, g6 and g8 are bayonet joints. Because of the varied piping elevation from the experimental hall of TLS to the building of the SRF laboratory, LHe supply lines L4–L12 and the CGHe return lines g2–g10 have cryogenic piping with ascending and descending sections. Figure 1 shows also the complicated piping path between VB #1 and VB #2, with details available in the article presented by Lin et al. (2010).

During the operation of the SRF module under test, the cryogenic valves at VB #1, for both LHe and CGHe, are operated in a manual mode with a fixed opening, typically fully open. The LHe valve at VB #2 regulates the LHe supply flow rate to maintain the SRF module with liquid at a constant level; the CGHe valve at VB #2 regulates the CGHe return flow rate from the SRF module to maintain constant its operational pressure at the LHe vessel. These two cryogenic regulating valves are controlled with independent controllers.

## Design process and test results of the cryogenic test stand

A 500-MHz superconducting cavity has a bell-shaped shell structure with nominal thickness 3 mm and is made of highly pure niobium; its mechanical structure is thus weak and soft. The warm cavity itself has a susceptibility to buckle. Taking the TLS 500-MHz SRF module as an example (shown in Fig. 1), its threshold buckling pressure is about 150–200 kPa (21.75–29 psia, 1500–2000 mbar) for the cavity when warm. This condition forces the operational pressure of a SRF module not to exceed 130.0 kPa (18.85 psia, 1300 mbar), but the routine operational pressure is further decreased to 126.2 kPa (18.3 psia = 1262 mbar) because of other operational concerns. The operating pressure of MD #1 is thus optimized at 140–150 kPa (1400–1500 mbar), even possibly to increase its operational pressure to 200 kPa, which is appreciably less than the critical point, 227.5 kPa. The fully loaded pressure drop must thus be within the range 13.8–23.8 kPa (1380–2380 mbar) for the LHe supply line from MD #1 to the SRF module of S1. The suction line of the first compressor, Comp #1, with warm gaseous helium returned from CB #1 has a pressure designed to be slightly above atmospheric pressure to avoid sucking air into the cryogenic plant, i.e. 105 kPa (1050 mbar). The CGHe return pressure drop of the heat exchangers inside CB #1 of medium size is about 15–20 kPa (150–200 mbar), dependent on the operational mass flow rate from the CGHe return line. CB #1 takes a pressure drop 17.5–19.0 kPa (175–190 mbar) when fully loaded; i.e. when both the on-duty and tested SRF modules are in operation concurrently. An acceptable pressure drop within 3.7–2.2 kPa is consequently reserved for the CGHe return line from the SRF module on duty back to the cold return port of CB #1. Such a tight constraint on the maximum allowable pressure drop creates extreme difficulty for the transfer of two-phase helium fluid if the cryogenic flexible transfer lines are long.

The performance of the 500-MHz SRF modules (Lo et al. 2013) of KEKB-type (B-factory accelerator at High Energy Accelerator Research Organization, Japan) for newly constructed synchrotron light source Taiwan Photon Source at NSRRC is examined at the cryogenic test stand in the SRF laboratory. The acceptance test includes maximum RF gap voltage 2.4 MV for a short-time operation of several minutes and RF gap voltage 1.6 MV for a test of long-term reliability over weeks. The dynamic load of a tested SRF module with a cavity quality factor 7.0 × 10^8^ at RF gap voltage 2.4 MV is about 90 W, and at RF gap voltage 1.6 MV is about 40 W. The KEKB-type SRF module can be operated only at a pressure not greater than 126.2 kPa (18.3 psia). Our objective is hence to design the cryogenic transfer lines to transfer the dynamic load 40 W to the KEKB-type SRF module operated at 126.0 kPa when the SRF module on duty at TLS operates routinely. As for the acceptance test with maximum RF gap voltage 2.4 MV, it is not an issue because it is just a short-time test and can be achieved in several ways without interfering with the operation of the SRF module on duty in TLS.

The 220-m flexible cryogenic transfer lines were originally designed with our simple analytical approach with the introduction of tolerance factor 1.5, applied to avoid a possible underestimate in the pressure drops of long cryogenic transfer lines. The maximum dynamic load available at the cryogenic test stand is about 60 W when the tested SRF module is operated at pressure 126.0 kPa after the experimental verification with the equivalent heater power. The KEKB-type SRF modules were successfully examined at RF gap voltage 1.6 MV over weeks; the impact on routine operation of the TLS SRF module is shown to be sustainable under the acceptance test. The maximum dynamic load available exceeds design objective 20 W, which implies that an actual tolerance factor for the 220-m flexible cryogenic transfer lines as built should be less than 1.5. It becomes verified on performing the experimental measurement for the accumulated static heat load of the LHe supply line.

On trying to impel the dynamic load of a tested SRF module up to 90 W, or even more, a phenomenon is observed. The extra pressure drop along the CGHe return line prevents sending the evaporated CGHe back to CB #1. After that, the LHe vessel pressure of the tested SRF module cannot be maintained and increases gradually, which prevents the delivery of LHe to the tested SRF module. Effective solutions have been found according to which, first, the operating pressure of MD #1 is increased to compensate the extra pressure drop from the LHe supply line, and, second, the speed of the turbines of the CB #1 is decreased (for example, slowing one step of speed so as to decrease the pressure drop of the heat exchangers inside CB #1 by about 5 kPa) or guiding partial CGHe flows directly to the suction line through the bypass line, so that less CGHe flows back to CB #1; the operating pressure of the tested SRF module can consequently stay at the expected condition. Both slowing the turbines of CB #1 and decreasing the quantity of the CGHe return to CB#1 can decrease the pressure drop of the heat exchangers inside CB #1 but at a cost of degrading the refrigeration capacity of CB #1. The LHe level of MD #1 might decline slowly whether or not the cooling capacity at the cryogenic test stand is greater than the refrigeration capacity of CB #1. For the acceptance test with maximum RF gap voltage 2.4 MV, we adopted to guide partial CGHe directly to the suction line to compensate the extra pressure drop from the CGHe return line to regulate the LHe vessel pressure operated at 126.0 kPa and to elevate the pressure of MD #1 from 140 to 146 kPa to compensate the extra pressure drop from the LHe supply line to balance the LHe level of the tested SRF module. During the test period, the decay of the LHe level of MD #1 is nearly imperceptible.

## Results of experimental measurements

During the measurements of the pressure drops, the contribution of the static heat load to the cryogenic transfer lines is invariably present; the mass flow rate induced by the static heat load of the LHe supply line is thus essential for the corresponding computation. The pressure drops related to the 220-m LHe and CGHe transfer lines are directly measurable with pressure transducers available in VB #1 and VB #2, indicated with diamond symbols in Fig. 1. The Venturi-type flow meters (Forster and Graber 1996) are located at the CGHe return line upstream of both VB #1 and VB #2, as shown in Fig. 1. The pressure transducers and Venturi-type flow meters on VB #1 and VB #2 and the electric power of a heater inside the LHe vessel of S0 were well calibrated before measurement. On maintaining constant both the level and pressure of the LHe vessel of S0 and varying the electric power of a heater inside the LHe vessel, both the total mass flow rate and the accumulated static heat load of the LHe supply line from MD #1 to S0, with static heat load of S0 included, can be deduced from the differential pressure of the CGHe flow read from the Venturi-type flow meter with the technique developed; refer to Lin et al. (2013). The range of the applied heater power is limited by several operating parameters, such as the pressure of MD #1, the pressure of S0, the openings of the LHe valves at VB #1 and of the CGHe valves at VB #1, the speed of turbines in CB #1 etc. The LHe supply valve directly after MD #1, the valves inside the SVB, and the cold return valve of the CB #1 are fixed openings. For every setting of a new heater power for S0, the corresponding total mass flow rate and pressure drops of the 220-m flexible cryogenic transfer lines are measured from the average readout of the Venturi-type flow meter at VB #2 and of the pressure transducers at VB #1 and VB #2 after the system attains a new thermal equilibrium. The duration to achieve another equilibrium state is much greater than what is taken for the measurement.

**Fig. 2:**
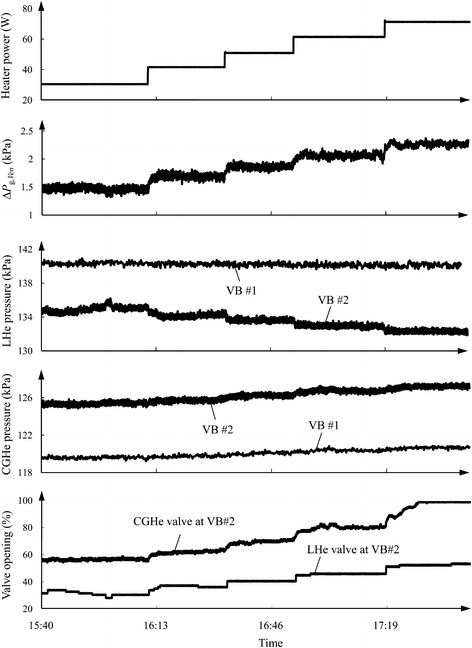
Under conditions of case 1, the differential pressure across the Venturi-type flow meter at VB #2 and the CGHe pressures at VB #1 and VB #2 increase when the electric power applied to the heater inside the LHe vessel of S0 is increased, but the LHe pressures at VB #1 and VB #2 decrease. The regulation valves of LHe and CGHe open gradually to compensate for the extra pressure drop caused by increased heater power

Table 1 lists the operating conditions of five test cases to be discussed here; Fig. 2 illustrates some measured results under the conditions of case 1. The varying range of level and pressure at a thermal equilibrium state when the heater power is applied in a range 30–70 W are about ±0.25% and ±0.075 kPa, respectively. As expected, differential pressure Δ*P*_g*,Ven*_ across a Venturi-type flow meter increases with increasing heater power, as the vaporized helium increases. MD #1, the LHe vessel of S0 and the suction line have constant pressures specified for all five test cases. The greater is the heater power, the greater must be the mass flow rate to maintain the LHe level constant, and the larger are the pressure drops along the cryogenic piping of both the LHe and CGHe transfer lines. As shown in Fig. 2, the LHe pressure drops between VB #1 and VB #2 increase with increasing heater power, but the trends of the LHe pressures at VB #1 and VB #2 decrease. The CGHe pressure drops between VB #1 and VB #2 increase with increasing heater power, and the trends of the CGHe pressures at VB #1 and VB #2 increase. At a large heater power, the valve resistance for the regulation valves must be decreased to compensate for the extra pressure drops along the cryogenic transfer lines. Eventually, the LHe valve at VB #2 opens wide to allow delivery of more LHe to S0, whereas the CGHe valve at VB #2 opens wide to allow sending more CGHe back to CB #1. The openings of LHe and CGHe valves at VB #2 are about 55.0 and 98.5% at heater power 70 W. The CGHe valve hence loses the function of pressure regulation when the heater power exceeds 70 W, which leads to an increased pressure in the LHe vessel of S0.

**Table 1:** Operating conditions and ranges of the applied heater power for five test cases

Case	MD #1 average pressure (kPa)	S0 average pressure (kPa)	LHe valve at VB #1 (%)	CGHe valve at VB #1 (%)	S0 heater power (W)
1	145	126.33	86.0	90	30–70
2	145	127.66	86.0	90	30–80
3	145	127.04	86.0	90	40–85
4	150	127.70	98.5	100	40–100
5	150	129.08	98.0	100	50–100

**Fig. 3:**
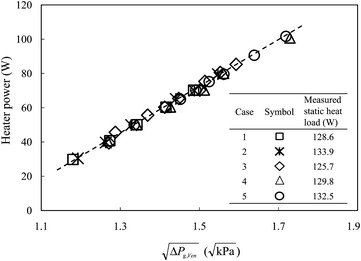
The electric power applied to the heater inside the LHe vessel of S0 exhibits a linear dependence on the square root of the measured differential pressure across the Venturi-type flow meter at VB #2. The accumulated static heat load along the LHe supply line is thus derived from a linear fit for these five test cases, i.e. the absolute value of the extrapolating heater power with a zero differential pressure drop read from the Venturi-type flow meter

**Fig. 4:**
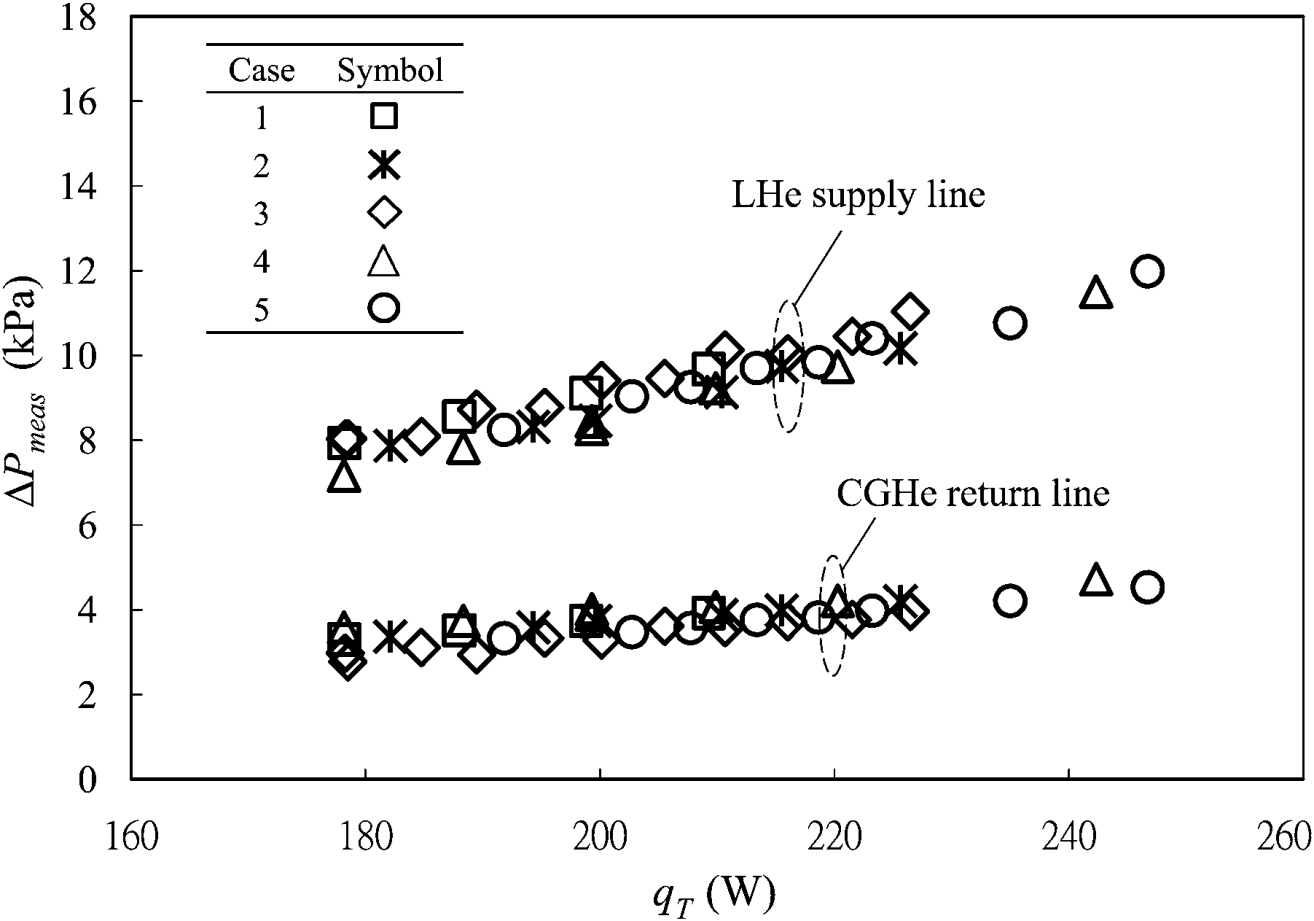
Measured pressure drops versus total heat loads for the LHe and CGHe transfer lines; the measured results show that these five test cases can be regarded as one group even though the operating conditions vary substantially

Figure 3 shows the measured differential pressures across the Venturi-type flow meter at VB #2 for the five test cases specified in Table 1. As expected, the applied heater power conforms to a linear fit with the square root of the differential pressure read from the Venturi-type flow meter. According to the technique developed (Lin et al. 2013), the accumulated static heat load along the LHe supply line from MD #1 to S0, with the static heat load of S0 included, are obtained from a linear-regression fit of each data set shown in Fig. 3. The accumulated static heat loads obtained for these five test cases are 128.6, 133.9, 125.7, 129.8 and 132.5 W, respectively. The relative discrepancy is less than 3.4% from average value 130.1 W, and is within the measurement tolerance. The flash loss, i.e. the gas vaporizing from the saturated liquid because of a pressure drop between MD #1 and S0, is less than 2% of the delivered LHe for these five cases; all measured data shown in Fig. 3 thus distribute near a single line even though the cryogenic operating conditions vary substantially. Total heat load *q*_*T*_ is a sum of the accumulated static heat load, the applied heater power and the flash loss; the relation of the total heat loads to the pressure drops is thus established. The measured pressure drops in the LHe and CGHe transfer lines for these five test cases are shown in Fig. 4. The data show that the pressure drops in the LHe and CGHe transfer lines are insensitive to the pressure drop between MD #1 and S0 and the openings of LHe and CGHe valves at VB #1 over this investigated range. The measured data are approximated with two separate straight lines, one for the LHe supply line and the other for the CGHe return line.

## Theoretical model for varied pressure drops

A theoretical model for pressure drops of various kinds of the piping elements of the cryogenic transfer lines shown in Fig. 1 follows. The pressure drop of an inclined pipe flow is induced mainly by both the friction between the fluids and the pipe wall and the elevation head; the latter is an effect of gravity from an altered elevation along the cryogenic piping path. The effects of the two-phase helium flow in the LHe supply line are treated with a simple theoretical model with consideration of the effect of gravity. Included also is the pressure drop caused by the resistance of cryogenic valves to the two-phase flow. Various pressure drops of the CGHe return line can be analyzed with the corresponding two-phase flow formula with vapor quality set to 1, whereas those of the assumed conditions on the LHe supply line with pure liquid are also computed with vapor quality set to 0. The LHe supply line is operated at a saturated state, but the CGHe return line is operated at a gaseous state.

### Inclined pipe pressure drop

For the LHe supply line, as mentioned above, a two-phase flow model must be considered, but, because of the complicated nature of two-phase flow, a simple theoretical model is introduced in our work for engineering applications from a practical point of view. Under the assumption of a steady, one-dimensional, incompressible and homogenous flow, the frictional and gravity pressure gradients for a two-phase flow in an inclined pipe with a uniform cross section (Collier 1981; Shannak 2008) satisfy

1 $$ - \left( {\frac{dP}{dz}} \right)_{(2\phi ),f} - \left( {\frac{dP}{dz}} \right)_{(2\phi ),s} = f_{(2\phi )} \frac{1}{{D_{h} }}\frac{1}{2}\frac{{\bar{G}_{{\left( {2\phi } \right)}}^{2} }}{{\rho_{(2\phi )} }} + \rho_{(2\phi )} \bar{g}\sin \theta $$

in which *z* is the direction along an inclined pipe and subscript $2\phi$ denotes two-phase flow. If the vapor quality of the two-phase flow is uniform along the inclined pipe, i.e. if the vapor quality is not a function of *z*, the frictional and gravity pressure drops for a two-phase flow, $\Delta P_{(2\phi ),f}$ and $\Delta P_{(2\phi ),s}$, are derivable from Eq. (1) as

2 $$ \Delta P_{(2\phi ),f} + \Delta P_{(2\phi ),s} = f_{(2\phi )} \frac{\ell }{{D_{h} }}\frac{1}{2}\frac{{\bar{G}_{{\left( {2\phi } \right)}}^{2} }}{{\rho_{(2\phi )} }} + \rho_{(2\phi )} \bar{g}\ell \sin \theta $$

with

3 $$ \bar{G}_{(2\phi )} = \rho_{(2\phi )} u_{(2\phi )} $$

in which $\ell$, *D*_*h*_ and *θ* are the length, hydraulic diameter and angle of inclination of the pipe, respectively; *f*, *ρ* and *u* are friction factor, density and velocity of the fluid in the pipe; $\;\bar{g}\;$ is the acceleration of gravity; $\bar{G}$ is the mass flux, defined as the product of density and velocity as written in Eq. (3). There exist several ascending and descending sections in the 220-m flexible cryogenic transfer lines; the pressure of a cryogenic fluid in an inclined pipe is thus affected by the variation of elevation of the cryogenic fluid. For example, the fluid flows upwards along the cryogenic piping, gaining elevation as it moves. The weight of fluid gradually decreases at the same time as there is less fluid above it. There is hence a loss of pressure as the fluid ascends along the cryogenic piping, and vice versa.

Because our simple theoretical model assumes a homogeneous flow, the two phases travel at the same velocity and behave as a single phase with the fluid properties defined as weighted averages of the properties of each individual phase. The density of the two-phase flow, $\rho_{(2\phi )}$, is defined as

4 $$ \frac{1}{{\rho_{(2\phi )} }} = \frac{x}{{\rho_{\text{G}} }} + \frac{{\left( {1 - x} \right)}}{{\rho_{\text{L}} }} $$

in which subscripts L and G denote saturated liquid and gaseous phases, respectively. The vapor quality, *x,* is defined as a ratio of vapor mass flow rate $\dot{m}_{\text{G}}$ to total mass flow rate $\dot{m}_{T}$, ($\dot{m}_{\text{G}} + \dot{m}_{\text{L}}$); in this work *x* is hence proportional to the accumulated static heat load along the LHe supply line. The vapor quality of cryogenic piping element *j*, $x_{j}$, is quantified after counting the static heat load along the LHe supply line *j* in our simple theoretical model of two-phase flow, i.e.

5 $$ x_{j} = \frac{{\sum\nolimits_{i = 1}^{j} {q_{{{\text{L}},calc}}^{(i)} } }}{{q_{T} }} $$

in which $q_{T}$ is the total heat load maintaining a level balance of liquid of S0; $q_{{{\text{L}},calc}}^{(i)}$ is the static heat load at cryogenic piping element *i* obtained from the engineering specification provided by vendors. In our case, there are in total 13 cryogenic piping elements in the LHe supply line from MD #1 to S0, shown in the upper part of Fig. 1. Note that the value *x* is constant at each cryogenic piping element.

When vapor quality *x* is determined, $\rho_{(2\phi )}$ is obtained from Eq. (4). The flow velocity is calculated from the mass flow rate equation, $\dot{m}_{T} = \rho_{(2\phi )} u_{(2\phi )} A$

6 $$ u_{(2\phi )} = u_{\text{L}} = u_{\text{G}} = \frac{{\dot{m}_{T} }}{{\rho_{ ( 2\phi )} \;A}} $$

in which *A* is the cross-sectional area of the pipe.

The two-phase friction factor $f_{(2\phi )}$ (Shannak 2008) is calculated with

7 $$ \frac{1}{{\sqrt {f_{(2\phi )} } }} = - 2\;\log \left[ {\frac{1}{3.7065}\left( {\frac{\varepsilon }{{D_{h} }}} \right) - \frac{5.0452}{{\text{Re}_{(2\phi )} }}\log \left( {\frac{1}{2.8257}\left( {\frac{\varepsilon }{{D_{h} }}} \right)^{1.1098} + \frac{5.8506}{{\left( {\text{Re}_{(2\phi )} } \right)^{0.8981} }}} \right)} \right] $$

in which *ε* is the surface roughness of the pipe; $\text{Re}_{(2\phi )}$ is the two-phase Reynolds number, defined as the ratio of the total inertial force of each phase to the total viscous force of each phase for a homogeneous two-phase flow (Shannak 2008). With respect to the definition of the Reynolds number of each phase, $\text{Re}_{\text{G}} = \bar{G}_{\text{G}} D_{h} /\mu_{\text{G}}$ and $\text{Re}_{\text{L}} = \bar{G}_{\text{L}} D_{h} /\mu_{\text{L}}$, in which *μ* is the dynamic viscosity of the fluid, the two-phase Reynolds number is expressed as

8 $$ \text{Re} _{{(2\varphi )}}  = \frac{{x^{2}  + \left( {1 - x} \right)^{2} \left( {{{\rho _{G} } \mathord{\left/ {\vphantom {{\rho _{G} } {\rho _{L} }}} \right. \kern-\nulldelimiterspace} {\rho _{L} }}} \right)}}{{\left( {{{x^{2} } \mathord{\left/ {\vphantom {{x^{2} } {\text{Re} _{G} }}} \right. \kern-\nulldelimiterspace} {\text{Re} _{G} }}} \right) + \left( {{{\left( {1 - x} \right)^{2} } \mathord{\left/ {\vphantom {{\left( {1 - x} \right)^{2} } {\text{Re} _{L} }}} \right. \kern-\nulldelimiterspace} {\text{Re} _{L} }}} \right)\left( {{{\rho _{G} } \mathord{\left/ {\vphantom {{\rho _{G} } {\rho _{L} }}} \right. \kern-\nulldelimiterspace} {\rho _{L} }}} \right)}} $$

The frictional and gravity pressure drops of the two-phase flow in an inclined pipe are thus calculated from the above equations.

### Valve pressure drop

The cryogenic regulating valves in VB #1 and VB #2 are commercial products (WEKA AG, Switzerland) and of equal-percentage type for both LHe and CGHe transfer lines, with maximum valve coefficient ($K_{{\nu ,\max }}$) 1.61 × 10^−4^ ${\rm m}^{3} /(s.\sqrt {\rm kPa} )$ (= 5.8 ${\rm m}^{3} /(h\sqrt {bar} )$ and rangeability $R = 2 0$. The pressure drop for a homogeneous two-phase flow through a valve (WEKA AG 2008), $\Delta P_{(2\phi ),val}$, is calculated as

9 $$ \Delta P_{{{\text{G}},val}}^{2} - P_{1} \;\Delta P_{{{\text{G}},val}} + \rho_{\text{g}} |_{{_{{(273{\text{K}},101.3{\text{kPa}})}} }} T_{1} \left( {\frac{{x\dot{V}_{\text{g}} |_{{(273{\text{K}},101.3{\text{kPa}})}} }}{{51.9K_{{\nu ,\max }} \;R^{{\bar{L}_{\text{G}} - 1}} }}} \right)^{2} = 0 $$

for the saturated gaseous phase, and

10 $$ \Delta P_{{{\text{L}},val}} = \frac{{\rho_{\text{L}} }}{1000}\left( {\frac{{(1 - x)\dot{V}_{\text{L}} }}{{K_{{\nu ,\max }} R^{{\bar{L}_{\text{L}} - 1}} }}} \right)^{2} $$

for the saturated liquid phase, in which $\dot{V}_{\text{g}} |_{{(273{\text{K}},101.3{\text{kPa}})}}$ is the equivalent volumetric flow rate of the gas at 273 K and 101.3 kPa, in unit m^3^/s; $\dot{V}_{\text{L}}$ is the volumetric flow rate of the saturated liquid, also in unit m^3^/s; $\rho_{\text{g}} |_{{(273{\text{K}},101.3{\text{kPa}})}}$ is the specific density of the gas at 273 K and 101.3 kPa, in unit kg/m^3^; $\rho_{\text{L}}$ is the specific density of the saturated liquid, also in unit kg/m^3^; $\bar{L}_{\text{G}}$ and $\bar{L}_{\text{L}}$ are the effective valve openings for the saturated gas and liquid, respectively, in %; $\Delta P_{{{\text{G}},val}}$ and $\Delta P_{{{\text{L}},val}}$ are the pressure drops of the valve, in unit kPa, contributed by the saturated gas and liquid, respectively; *P*_1_ is the upstream pressure of the valve in kPa, and *T*_1_ is the upstream temperature of the valve in K. Physically, $\Delta P_{{{\text{G}},val}}$ and $\Delta P_{{{\text{L}},val}}$ have the same value; the sum of $\bar{L}_{\text{G}}$ and $\bar{L}_{\text{L}}$ must match the measured valve opening, $\bar{L}_{ ( 2\phi )}$; Eqs. (9) and (10) are solved iteratively to fulfill these two conditions. Equation (9) with vapor quality *x* = 1 is solvable for the valve pressure drop of the pure gas; Eq. (10) with vapor quality *x* = 0 is solvable for the valve pressure drop of the pure liquid.

## Heat loads of the cryogenic transfer lines

### General assumptions and working parameters

To verify the results obtained from the simple analytical approach with the measured pressure drops, we applied the theoretical formulae previously deduced to the related calculations. Some assumptions and conditions applied to simplify the computation follow.

1. We suppose that the entire upstream heat load of transfer lines L1 to L4 flows to S0.
2. The fluid pressure is approximated as a constant value in the LHe and CGHe transfer lines to calculate the required properties, viscosity and density, at each cryogenic piping element. The pressure at S0 is assigned to be the common pressure of the LHe supply lines from L1 to L13; the return pressure of CB #1, *P*_*CB#*1_, measured downstream from the SVB is assigned to be the common pressure of the CGHe return lines from g1 to g13.
3. The surface is assumed to be smooth for cryogenic transfer lines L4, L6, L8, L10, L12, g2, g4, g6, g8 and g10 that are made of non-corrugated pipes (solid tubes with smooth surfaces) with the corresponding friction factor calculated with Eq. (7).
4. Another way to obtain the surface roughness *ε* of a corrugated pipe is available in the literature (Laeger et al. 1978; Jaiman et al. 2010; Uslu and Ahn 2013). We chose estimated value 0.08 for our calculation in corrugated pipes according to the paper (Laeger et al. 1978) for a fluid with Reynolds number about 10^5^, as both LHe and CGHe transfer lines, L5, L7, L9, L11, g3, g5, g7 and g9, have Reynolds numbers between 1 × 10^5^ and 4 × 10^5^ for all simulated cases.
5. The effect of an elbow, a bend of the corrugated pipe, a sudden enlargement and a sudden contraction on the pressure drop are also considered. For their loss coefficient, *K*_*L*_, we referred to the literature (Munson et al. 2001). The values for the 90° elbow, common elbow and corrugated bends with *ε* = 0.08 are 1.1, 0.3 and 0.65, respectively.
6. The pressure drop across a Venturi-type flow meter is typically between 10 and 20% of the measured differential pressure (Forster and Graber 1996); a pressure drop 15% of the measured differential pressure of the Venturi-type flow meter at VB #2 is thus added to the calculated pressure drop for the CGHe return line to simulate the pressure drop across the Venturi-type flow meter at VB #1.
7. The outlet temperature from the LHe vessel is assumed to be 5 K. The saturated temperature at the LHe vessel is about 4.5 K for these five test cases.

### Two-phase LHe supply line

According to the specifications of each LHe supply line, the engineering static heat load of each cryogenic piping element is listed in Table 2. The accumulated static heat load 72.34 W is thus calculated from

11 $$ q_{{{\text{L}},calc}} = \sum\limits_{i = 1}^{N} {q_{{{\text{L}},calc}}^{(i)} } $$

in which *N* = 13 is the total number of cryogenic piping elements involved in calculating the heat load, shown in the upper part of Fig. 1. All values of the engineering static heat loads are, however, approximate, used for system evaluation during the design phase. The measured accumulated static heat load along the LHe supply line from MD #1 to S0, $q_{{{\text{L}},meas}}^{{({\text{total}})}}$, is listed in the table in Fig. 3 for the five test cases with varied operating conditions, in which the measured static heat load to S0, $q_{{{\text{L}},meas}}^{{({\text{S0}})}}$ = 37 W (Chang et al. 2011), is embedded. The measured accumulated static heat load of entire LHe supply line is obtained as

12 $$ q_{{{\text{L}},meas}} = q_{{{\text{L}},meas}}^{{({\text{total}})}} - q_{{{\text{L}},meas}}^{{({\text{S0}})}} $$

The measured accumulated static heat load from MD #1 to S0 has average value 130.1 W. The measured accumulated static heat load of entire LHe supply line is thus deduced as 93.1 W. Tolerance factor *F*_*B*_ is here defined as the ratio of the measured average result to the engineering accumulated static heat load,

13 $$ F_{B} = \frac{{q_{{{\text{L}},meas}} }}{{q_{{{\text{L}},calc}} }} $$

A considerable deviation of heat load between the designed and the post-measured performance of cryogenic transfer lines might occur for various reasons, for example interfaces between individual cryogenic components, an inappropriate piping path from constraints in civil engineering, assembly defects from human negligence etc., which depend strongly on the details of the specific application but are less relevant to the general performance of cryogenic components indicated in a engineering specification. As a common rule of thumb adopted for cryogenic designs, safety factor 1.5 is taken to be tolerance factor 1.5 in designing a cryogenic transfer lines as a design margin in the design phase. Tolerance factor 1.5 accounts for these uncertainties so as to avoid an underestimate at the pressure drops of cryogenic transfer lines at the cost of increased facility costs. Tolerance factor *F*_*B*_ given herein is to reflect the deviation defined as the ratio of the measured average result to the engineering accumulated static heat load. Unit *F*_*B*_ = 1.0 representing the actual accumulated static heat load is fully consistent with the sum of engineering specifications provided by vendors. For most cases, the actual accumulated static heat load is greater than the engineering specification, i.e. *F*_*B*_ is greater than 1. The significance of tolerance factor *F*_*B*_ in our analysis comes from two considerations: first, it reflects the quality of construction of a specific cryogenic transfer line because it includes all construction uncertainties of the heat load, and, second, it allows the flexibility required to accommodate the possible inaccuracies between the actual and the engineering accumulated static heat loads. It can therefore serve to verify the validity of the theoretical tools of a pressure-drop calculation for design work.

**Table 2:** List of type, length, inner diameter (regarded as hydraulic diameter) and heat-load specification for each cryogenic piping element of the LHe supply line. Note that the vacuum-jacketed solid line of L1 includes two vertical bayonets; the multi-channel line of L1 includes one cryogenic value

Element	L1		L2	L3	L4	L5	L6	L7	L8	L9	L10	L11	L12	L13
Type	+	■	+	■	+	○	Horizontal bayonet	●	Horizontal bayonet	●	Horizontal bayonet	○	+	○
Size [ℓ (m), D_h_ (mm)]	(3.1,22.45)	(3.8,22.45)	(1.5,22.45)	(4,22.45)	(1,13.85)	(5,20.2)	(0.6,22.45)	(67,21)	(0.6,22.45)	(138,21)	(0.6,22.45)	(2.5,17.12)	(1,17.12)	(8,27.86)
Heat load (W)	8.95	1.14	6.1	1.2	1.4	7.5	4.75	4.02	4.75	8.28	4.75	3.5	1.4	14.6

+ vacuum-jacketed solid line; ■ multi-channel line; ○ vacuum-jacketed flexible line; ● four-tube flexible coaxial line

In our case, tolerance factor *F*_*B*_ is thus obtained as 93.1/72.34 = 1.287. The measured accumulated static heat load of the entire LHe supply line is hence 28.7% greater than the sum of the specified values provided by the vendors, which is still less than commonly adopted safety factor 1.5 for cryogenic-related designs. Being unable to identify the actual tolerance factor element by element of the cryogenic piping, we considered it reasonable to proceed to multiply this tolerance factor *F*_*B*_ by the engineering static heat load of each piping element of the LHe supply line to approach the measured accumulated static heat load. Given the heat load of the LHe supply line, the various pressure drops are obtainable according to the theoretical model given in “Theoretical model for varied pressure drops” section. The information related to each cryogenic piping element for the calculation is also listed in Table 2.

The vapor quality for each cryogenic piping element is then calculated as

14 $$ x_{j} \cong \frac{{F_{B} \left( {\sum\nolimits_{i = 1}^{j} {q_{{{\text{L}},calc}}^{(i)} } } \right)}}{{\left( {1 + X} \right)\left( {q_{{{\text{L}},meas}}^{{({\text{total}})}} + q_{h} } \right) + q_{{{\text{L}},meas}}^{{({\text{S1}})}} }},\quad j = 1  \sim 4 $$

and

15 $$ x_{j} \cong \frac{{F_{B} \left( {\sum\nolimits_{i = 1}^{j} {q_{{{\text{L}},calc}}^{(i)} } } \right)}}{{\left( {1 + X} \right)\left( {q_{{{\text{L}},meas}}^{{({\text{total}})}} + q_{h} } \right)}},\quad j = 5 \sim N $$

with parameter *Χ* of the flash ratio defined (Lin et al. 2013) as

16 $$ X = \frac{{h_{\text{L}}^{{({\text{MD}}\# 1)}} -
h_{\text{L}}^{{({\text{S}}0)}} }}{{h_{\text{G}}^{{({\text{S}}0)}} -
h_{\text{L}}^{{({\text{S}}0)}} }} $$

in which $q_{h}$ is the applied heater power at S0; $h_{\text{L}}^{{({\text{MD}}\#\,1)}}$ is the enthalpy of saturated LHe at MD #1 of which the value is determined only by the pressure of MD #1; $h_{\text{L}}^{{({\text{S0}})}}$ and $h_{\text{G}}^{{({\text{S0}})}}$ are enthalpies of saturated liquid and gaseous helium at S0, respectively, of which their values depend also on only the pressure of S0. Total heat load S1, $q_{meas}^{{({\text{S1}})}}$, is approximately 65 W, as a sum of static heat load 30 W (Lin et al. 2006) and applied heater power 35 W. This heat load is thus included in the total heat load for all test cases to calculate the pressure drops of the long cryogenic transfer lines. This heat load is critical in calculating the pressure drops of LHe supply lines L1 to L4 and CGHe return lines g10 to g13, but has a minor effect on the other cryogenic transfer lines. As L4 and g10 are both short sections of the 220-m flexible cryogenic transfer lines, the heat load due to the operation of S1 causes a minor effect on the calculations of the pressure drops of the 220-m flexible cryogenic transfer lines.

When vapor quality *x* is determined, the total pressure drop of the LHe supply line between VB #1 and VB #2 is calculated with

17 $$ \Delta P_{L,calc} = \sum\limits_{i = 4}^{11} {\Delta P_{(2\phi ),f}^{(i)} } + \sum\limits_{i = 4}^{11} {\Delta P_{(2\phi ),s}^{(i)} } + \Delta P_{(2\phi ),val} $$

According to the positions of the installed pressure sensors, only the effects of the valves at VB #1 are included in calculating the pressure drops of the 220-m flexible cryogenic transfer lines. Additionally, the pressure drops contributed from the elbows, bends of corrugated pipes, sudden enlargements and sudden contractions in each cryogenic piping element are included in the frictional pressure drop, $\Delta P_{ ( 2\phi ),f}^{(i)}$, in our calculation.

### CGHe return line

The temperature rise of the CGHe return line due to the heat load is calculated from the enthalpy rise at each cryogenic piping element as

18 $$ h_{j} = \frac{{\dot{m}_{T} h_{\text{g}}^{{({\text{S0}})}} + \sum\nolimits_{i = 1}^{j} {F_{B} q_{{{\text{g}}, \, calc}}^{(i)} } }}{{\dot{m}_{T} }},\quad j = 1 \sim 10 $$

in which $q_{{{\text{g}}, \, calc}}^{(i)}$ is the engineering static heat load at cryogenic piping element *i* listed in Table 3; $\dot{m}_{T}$ is the total mass flow rate; $h_{\text{g}}^{{({\text{S0}})}}$ is the enthalpy determined according to the property pair *P*_S0_ and 5 K. Tolerance factor *F*_*B*_ for the two-phase LHe supply line is applied also to the CGHe return line to simulate the actual static heat load.

**Table 3:** List of type, length, inner diameter (regarded as hydraulic diameter) and heat-load specification for each cryogenic piping element of the CGHe return line

Element	g1	g2	g3	g4	g5	g6	g7	g8	g9	g10
Type	○	+	○	Horizontal bayonet	●	Horizontal bayonet	●	Horizontal bayonet	○	+
Size [ℓ (m), D_h_ (mm)]	(8,38.2)	(1,27.86)	(2.5,27.86)	(0.6,22.45)	(138,38.9)	(0.6,22.45)	(67,38.9)	(0.6,22.45)	(5,40.1)	(1,22.45)
Heat load (W)	17.8	2.8	4.75	4.75	16.56	4.75	8.04	4.75	11.5	2.8

+ vacuum-jacketed solid line; ○ vacuum-jacketed flexible line; ● four-tube flexible coaxial line

We used commercial software (HEPAK, Cryodata Inc.) to compute the required properties of gaseous helium, density and viscosity, at each cryogenic piping element according to property pair *P*_*CB#*1_ and *h*_*j*_. Together with the geometric information listed in Table 3, the pressure drops of the cryogenic piping element from g3 to g10, as shown in the upper part of Fig. 1, were then calculated. The total pressure drop of the CGHe return line between VB #2 and VB #1 is obtained as

19 $$ \Delta P_{{{\text{g}},calc}} = \sum\limits_{i = 3}^{10} {\Delta P_{{{\text{g}},f}}^{(i)} } + \sum\limits_{i = 3}^{10} {\Delta P_{{{\text{g}},s}}^{(i)} } + \Delta P_{{{\text{g}},val}} + \Delta P_{{{\text{g}},Ven}} $$

in which $\Delta P_{{{\text{g}},f}}^{(i)}$ and $\Delta P_{{{\text{g}},s}}^{(i)}$ are the pressure drops of CGHe piping element *i* due to effects of friction and gravity, respectively; $\Delta P_{{{\text{g}},val}}$ is the pressure drop of the CGHe valve at VB #1; $\Delta P_{{{\text{g}},Ven}}$ is the pressure drop induced by the Venturi-type flow meter at VB #1. These pressure drops are obtainable from the corresponding two-phase flow formula with unit vapor quality. The pressure drops contributed from the elbows, bends of corrugated pipes, sudden enlargements and sudden contractions in each cryogenic piping element are included in the frictional pressure drop, $\Delta P_{{{\text{g}},f}}^{(i)}$, in our calculation.

## Results and discussion

### Verification of theoretical model for frictional pressure drop

The two-phase frictional multiplier term *Φ* (Rane et al. 2011) is defined as

20 $$ \varPhi^{2} = \frac{{\Delta P_{(2\phi ),f} /\ell }}{{\Delta P_{(1\Phi ),f} /\ell }} $$

With

21 $$ \Delta P_{(1\phi ),f} = f_{(1\phi )} \frac{1}{{D_{h} }}\frac{1}{2}\frac{{\bar{G}_{(1\phi )}^{2} }}{{\rho_{(1\phi )} }} $$

in which $\Delta P_{(1\phi ),f}$ is the frictional pressure drop for a single-phase flow. Here a single-phase flow means that pure liquid flows along a pipe. Using Eqs. (2), (3) and (6) in Eq. (20), we obtain

22 $$ \varPhi^{2} = \frac{{f_{(2\phi )} \rho_{(1\phi )} }}{{f_{(1\phi )} \rho_{(2\phi )} }} $$

**Fig. 5:**
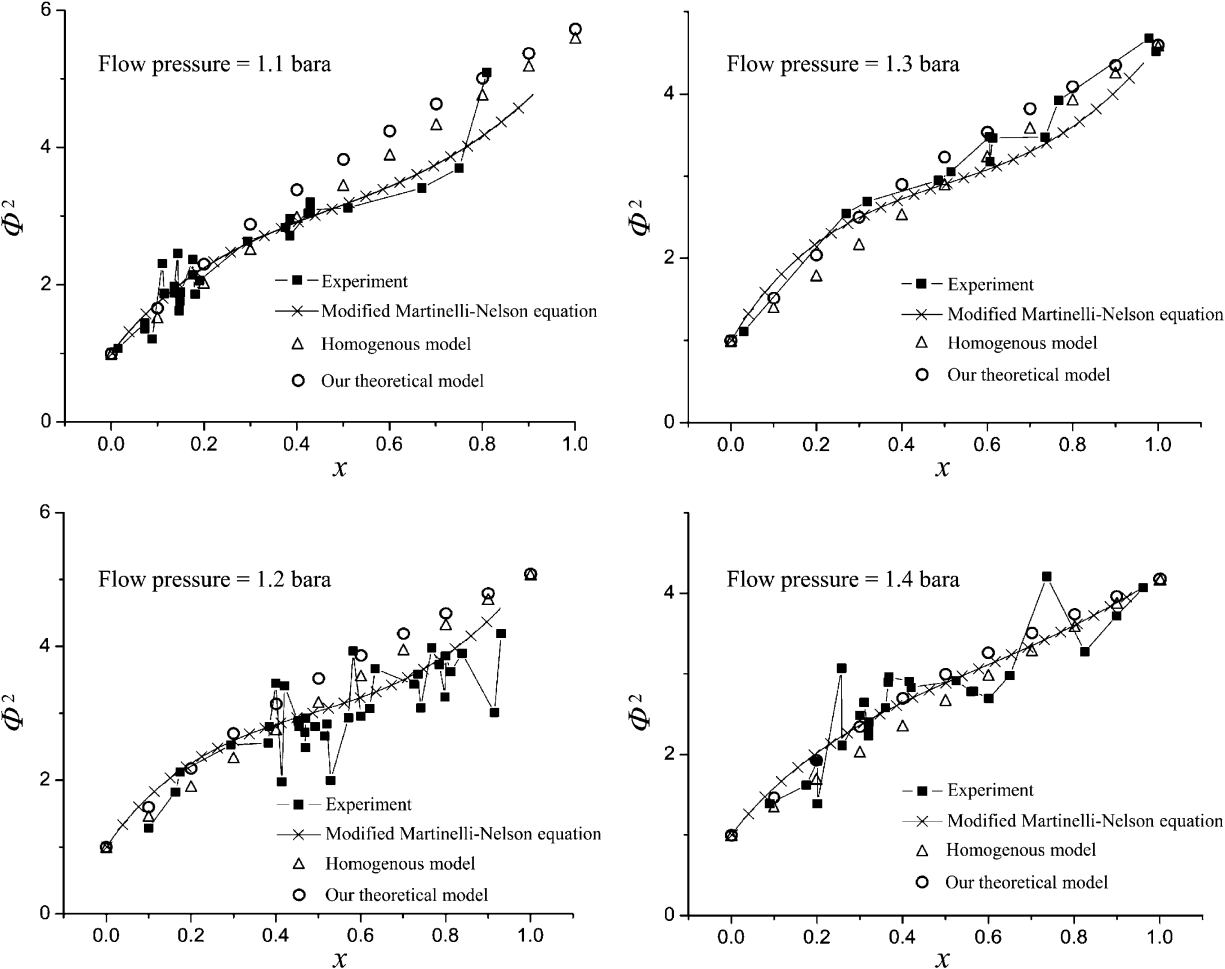
Calculated results of two-phase frictional multiplier term *Φ* ^2^ varying with vapor quality × with our theoretical model are compared with the experimental data, the modified Martinelli–Nelson equation and the homogenous model (Rane et al. 2011) (Courtesy Cryogenics 2011)

The accuracy of frictional pressure drops in a horizontal pipe with a smooth surface calculated with our theoretical model of two-phase helium flow is verified with available experimental data (Rane et al. 2011) and other theoretical models, for example, the homogenous flow model (Collier 1981) and the Martinelli–Nelson equation with improved correlation term (Rane et al. 2011). Figure 5 shows a comparison for calculated *Φ* ^2^ vs vapor quality *x* at flow pressures 1.1, 1.2, 1.3 and 1.4 bar. The rectangles with connecting line, crosses with connecting line, triangles and circles represent the data obtained from experiment, the Martinelli–Nelson equation with an improved correlation term, the homogenous flow model and our theoretical model, respectively. For our case, the operating pressure in the LHe supply line is in a range 1.5–1.26 bara. The operating range of the total heat load (i.e. total mass flow rate) is 180–250 W based on our requirement. The accumulated static heat load from MD #1 to the location of VB #2 with *F*_*B*_ = 1.287 is 74.3 W (deduced from Table 2); the minimum applied heat load is 180 W; the applied maximum vapor quality *x* is hence derived as 0.41. From the comparison plots, the discrepancy of calculated results, for *x* less than 0.41, between our theoretical model and experimental data is found to be acceptable for each flow pressure discussed in Fig. 5. This result is a demonstration that our theoretical model is suitably applicable to the frictional pressure-drop calculation of the bubble regime of flow in flow pressure range 1.1–1.4 bara. In particular, when the flow pressure is between 1.3 and 1.4 bara, our theoretical model is applicable also to all regimes of flow with acceptable error. The LHe supply line is commonly designed with the aim of a small heat load; the maximum vapor quality for most cases with long cryogenic transfer lines is thus still maintained small so that the frictional pressure drop is calculable with our theoretical model. The next investigation is whether the theoretical model is applicable to the frictional pressure-drop calculation in the CGHe return line. The calculated results of pure gas (i.e. *x* = 1) in the CGHe return line are compared also with the experimental data in flow pressure range 1.1–1.4 bara. For our case, the operating pressure in the CGHe return line is in the range 1.26–1.15 bara. The experimental data are unavailable at *x* = 1 for pressures 1.1 and 1.2 bara, but the calculated results for pressures 1.3 and 1.4 bara agree approximately with the experimental data.

### Two-phase LHe supply line

**Fig. 6:**
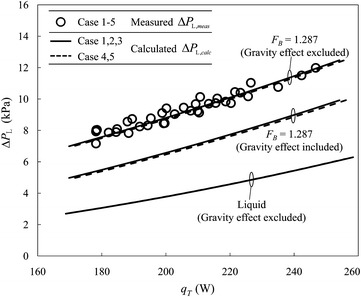
With tolerance factor *F*_*B*_ = 1.287 applied, the calculated total pressure drop of the 220-m LHe supply line between VB #1 and VB #2 in NSRRC approaches the measured results much better when the effect of gravity is neglected

The measured, $\Delta P_{{{\text{L}},meas}}$, and calculated, $\Delta P_{{{\text{L}},calc}}$, total pressure drops of the LHe supply line between VB #1 and VB #2 as functions of the total heat load, $q_{T}$, are illustrated in Fig. 6. Tolerance factor *F*_*B*_ = 1.287 has been applied to the calculated results shown for a best match with the measured results. The calculated results for the five test cases with varied operating conditions are divided into two groups, one for cases 1, 2 and 3 with LHe valve opening 86% at VB #1, and the other for cases 4 and 5 with LHe valve openings 98 and 98.5% at VB #1; the two groups are marked with solid and dashed lines, respectively. The opening of the LHe valve at VB #1 evidently affects the group behaviour, but gap 0.1 kPa between these two groups is still within the fluctuation of the measured data. The openings of LHe and CGHe valves at VB #2 are highly sensitive to the mass flow rate, but the corresponding valve pressure drops do not contribute to the measured pressure drops of the LHe and CGHe transfer lines between VB #1 and VB #2.

Mean error (ME) and mean percentage error (MPE) are defined as

23 $$ {\text{ME}} = \sum\limits_{i = 1}^{M} {(\Delta P_{{{\text{L}},calc}}^{(i)} - \Delta P_{{{\text{L}},meas}}^{(i)} )} /M $$

24 $$ {\text{MPE}} = \sum\limits_{i = 1}^{M} {((\Delta P_{{{\text{L}},calc}}^{(i)} - \Delta P_{{{\text{L}},meas}}^{(i)} )} /\Delta P_{{{\text{L}},meas}}^{(i)} )/M \times 100_{{}} \% $$

in which *M* denotes the number of experimental data. The positive values of ME and MPE represent overestimates whereas the negative values represent underestimates. The values of ME and MPE tend to zero, which indicates that the calculated results match satisfactorily the experimental results. Taking cases 1, 2 and 3 in Fig. 6 for example, a linear fit of the measured total pressure drops is $\Delta P_{{{\text{L}},meas}}$[kPa] = 0.0603 ×$q_{T}$[W] − 3.112, whereas $\Delta P_{{{\text{L}},calc}}$[kPa] = 0.0587 ×$q_{T}$[W] − 5.1026 for the calculated results when the net effect of gravity due to elevation difference 4.72 m of the LHe supply line is included. As VB #1 is located 4.72 m above VB #2, gravity is theoretically expected to benefit the operation of the LHe supply line. The relative slope discrepancy is only 2.7%, but ME and MPE between the individual measured and calculated total pressure drops are −2.31 kPa and −24.7%, respectively. In conclusion, the least-square-fitted lines of the calculated results have a slope that matches well that of the measured data, but the calculated pressure drops are systematically less than the measured data with a considerable offset when the effect of gravity is considered. In contrast, when the effect of gravity is neglected, the calculated result approaches the measured pressure drop. The linear equation for the calculated results becomes $\Delta P_{{{\text{L}},calc}}$ = 0.065×$q_{T}$[W] − 4.1443 for cases 1, 2 and 3. The slope discrepancy for the fitted equation of the measured data increases to 7.8%, but ME and MPE between the individual measured and calculated total pressure drops decrease to −0.06 kPa and −0.7%, respectively. Comparison of these two results shows that the magnitude of frictional pressure drop for the two-phase LHe supply line is underestimated with the simple theoretical model. We conclude that gravity affects the interaction between the two phases of the two-phase flow and consequently varies its net effects on its frictional pressure drop. For example, the gas of a two-phase flow tends to float upwards, being mixed with the liquid because of the density difference, thus resulting in extra friction, especially when the flow direction is downwards. As a result, the benefit of the effect of gravity for the descending section of LHe supply line decreases. Extra friction of this kind induced from gas-phase buoyancy transport in the ascending and descending sections of the 220-m flexible two-phase LHe supply line is not taken into account in our simple theoretical model at present. For complicated piping of this kind with an altered elevation along the cryogenic piping path, especially for the case that gravity benefits the flow, it is suggested to neglect the benefit of gravity in the design phase to avoid an underestimate in this simple theoretical model of two-phase flow, as adopted in our present analysis.

A quick estimate of the pressure drops of the pure liquid on the LHe supply line between VB #1 and VB #2 with the same mass flow rate was made, but Fig. 6 shows that the pressure drops were significantly underestimated. This effect illustrates that the oversimplified method of estimation is unsuitable to calculate the pressure drop of the two-phase LHe supply line.

### CGHe return line

**Fig. 7:**
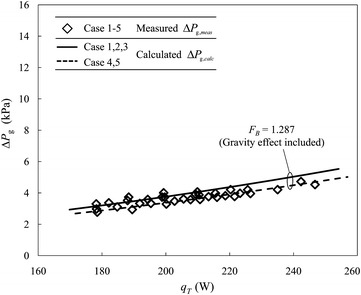
The calculated total pressure drop of the 220-m CGHe return line between VB #2 and VB #1 in NSRRC approaches the measured results with tolerance factor *F*_*B*_ = 1.287 applied

The measured and calculated total pressure drops, $\Delta P_{{{\text{g}},meas}}$ and $\Delta P_{{{\text{g}},calc}}$, of the CGHe return line between VB #1 and VB #2 as functions of the total heat load, $q_{T}$, are illustrated in Fig. 7. For the calculations, not only tolerance factor *F*_*B*_ = 1.287 is applied, but also the effect of gravity is considered as the fluid is a gas rising from VB #2 to VB #1. The calculated data are again divided into two groups, one for cases 1, 2 and 3 with the CGHe valve at VB #1 operated at opening 90%, the other group for cases 4 and 5 with opening 100%. The calculated results for cases 4 and 5 show a maximum gap 0.56 kPa at 250 W with the results for cases 1, 2 and 3 due to the varied opening of the CGHe valve at VB #1.

An improved agreement between the measured and calculated results is obtained for the gaseous flow, as shown in Fig. 7. The straight-line fit for the measured pressure drops of cases 1–5 has slope 0.0244 kPa/W and a constant term −1.351 kPa, for comparison with 0.0308 kPa/W and −2.3579 kPa for cases 1–3 and 0.0274 kPa/W and −2.07 kPa for cases 4 and 5, for the calculated results. The relative errors of the slope between the measured and calculated results for these two groups are 26.2 and 12.3%, respectively. ME and MPE between the individual measured and calculated total pressure drops are 0.29 kPa and 7.9% for cases 1–3 and −0.11 kPa and −3.2% for cases 4 and 5 within the applied total heat-load range, 180–250 W. If the valve pressure drop is neglected, the slope of the calculated total pressure drop becomes 0.0224 kPa/W for cases 1 to 3; the error of the slope decreases from 26.2 to 8.2%. These data reveal that the slope of the calculated total pressure drops is affected mainly by the valve pressure drop calculated from the empirical formula of the gas valve in Eq. (9).

**Fig. 8:**
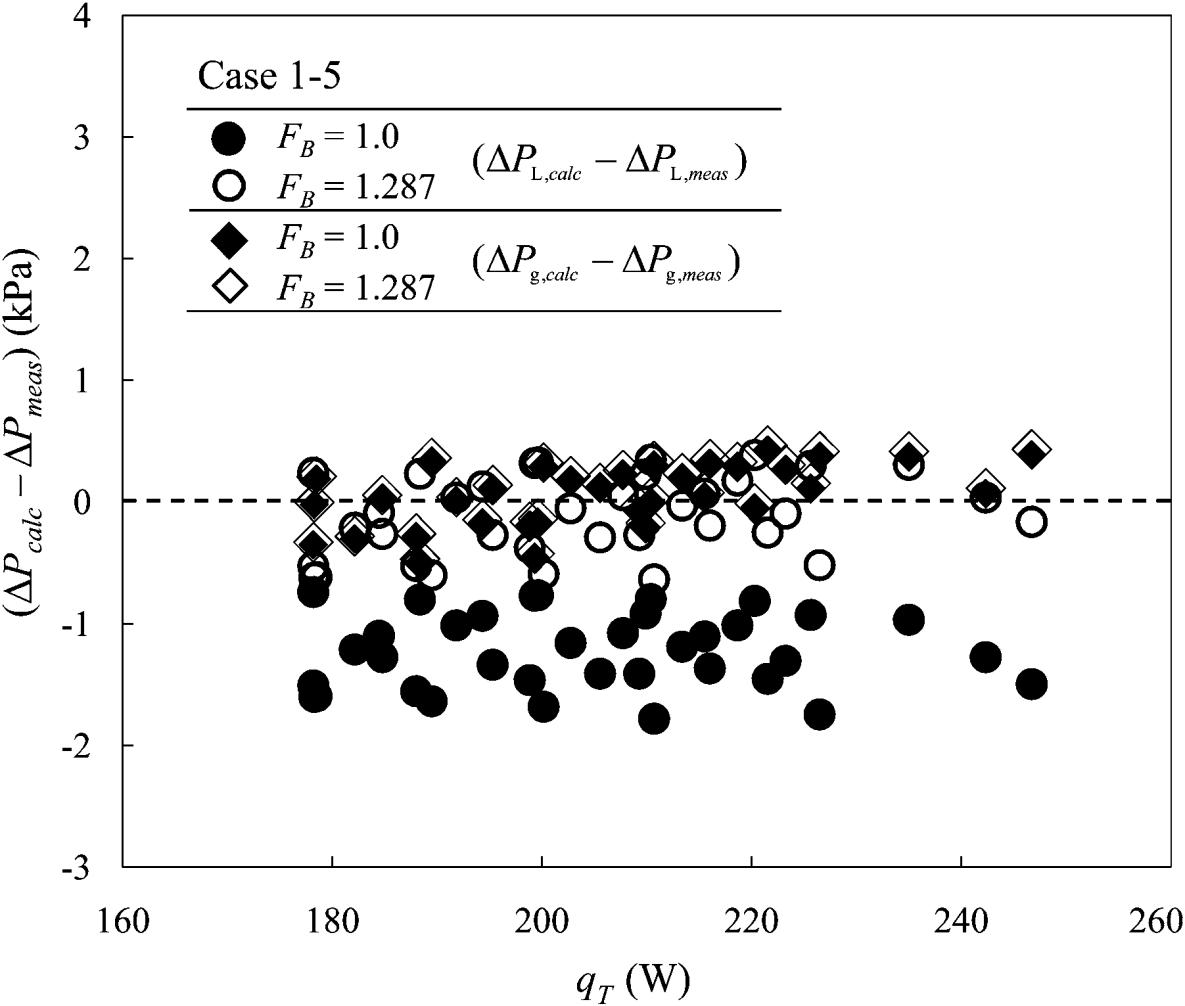
For cases 1 to 5 with tolerance factor *F*_*B*_ = 1.287 applied, the calculated total pressure drop for the two-phase LHe supply line approaches the measured results much better, but increases little the results for the CGHe return line

Figure 8 shows the differences between the measured and calculated total pressure drops for the 220-m flexible cryogenic transfer lines with tolerance factors *F*_*B*_ = 1.0 and 1.287 applied. Under the same total heat load, i.e. the same total mass flow rate, a greater *F*_*B*_ implies a greater heat load on the transfer line but a smaller heat load on the terminal LHe vessel; more gas inside the LHe supply line thus generates a greater pressure drop of a two-phase flow. For the pressure-drop calculations, the effect of gravity is taken into consideration for the CGHe return line, but not for the two-phase LHe supply line. For cases 1–5 with tolerance factor *F*_*B*_ = 1.287 applied, the calculated total pressure drop approaches the measured results; ME alters from −1.23 to −0.11 kPa. Applying tolerance factor *F*_*B*_ = 1.287 increases little the results for the CGHe return line as compared with the case with *F*_*B*_ = 1.0; ME is altered from 0.06 to 0.09 kPa, as the extra heat increases only slightly the temperature of the gas, and results in a slightly decreased density in the range of the mass flow rate 180–250 W. This condition illustrates that the simple analytical approach can estimate satisfactorily the pressure drop for the CGHe return line even if tolerance factor *F*_*B*_ = 1.0 is applied; the tolerance factor has only a minor effect on the pressure drop in the CGHe return line.

The actual tolerance factor is unknown in the design phase; the validity of a sufficient safety margin using tolerance factor 1.5 is demonstrated with the measured results of the 220-m flexible cryogenic transfer lines as built at NSRRC. The calculated result of cases 1 to 5 for the two-phase flow in the LHe supply line neglecting the effect of gravity has an overestimate 0.76 kPa relative to measured results; similarly, the tolerance factor has a minor effect on the pressure drop in the CGHe return line. As a result of these comparisons, value 1.5 of the tolerance factor is explained to suit well the present long-distance helium transfer line in the design phase.

### Remarks

**Fig. 9:**
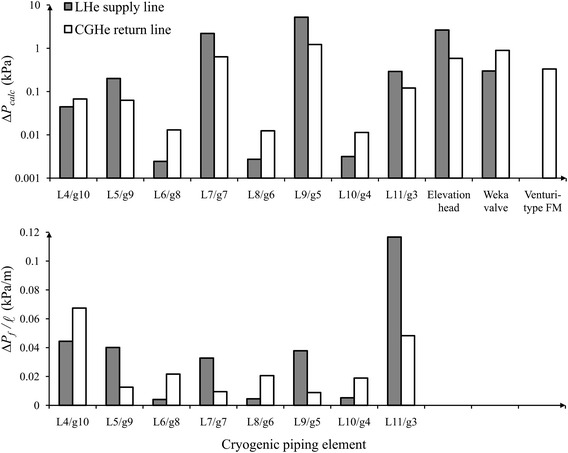
Pressure drops of individual cryogenic piping elements and corresponding pressure drops per unit length at total heat load 210 W for cases 1 to 3. The critical path for the pressure drop is revealed

For a detailed examination of the critical path for the pressure drop obtained from the calculations, illustrated in Fig. 9 are the pressure drops of individual cryogenic piping elements and the corresponding pressure drops per unit length at total heat load 210 W for cases 1 to 3. The upper plot of Fig. 9 shows that the long transfer sections of concentric lines of four-tube type, L7 and L9 for the LHe supply line, as well as g7 and g5 for the CGHe return line, contribute most frictional pressure drop, whereas the valves at VB #1, elevation head and Venturi-type flow meter also contribute a large pressure drop at this flow rate. The elevation head for the contribution of the LHe supply line is negative, i.e. an increased static pressure, but is plotted with its absolute value to show its magnitude on the total pressure drop. As shown in the lower plot of Fig. 9, as transfer sections L5, L7, L9 and L11 for the LHe supply line and g3 for CGHe return line have a greater frictional pressure drop per unit length, their increased diameters can be taken into account to decrease the frictional pressure drop if necessary. The bayonet joints of largest available size, effective diameter 22.45 mm, have been used to connect the long LHe and CGHe transfer lines. Bayonet joints g4, g6 and g8 for the CGHe return line have consequently a greater frictional pressure drop per unit length, but bayonet joints L6, L8 and L10 for the LHe supply line have a smaller frictional pressure drop per unit length.

## Conclusions

A cryogenic transfer system including flexible cryogenic transfer lines of corrugated type of total length 220 m has been installed at NSRRC (Lin et al. 2010) to deliver LHe for a cryogenic test stand. This system might be the longest cryogenic flexible lines ever implemented for similar applications. A successful design of cryogenic transfer lines has been made to provide the demanded cooling capacity at the cryogenic test stand based on our simple analytical approach with tolerance factor 1.5 in the estimate of pressure drops of the cryogenic transfer lines. The objective of a long-term reliability test of a KEKB-type SRF module examined at gap voltage 1.6 MV over weeks when the SRF module on duty at TLS operates routinely was also achieved. The test result proves also that value 1.5 of tolerance factor suits well a long-distance helium transfer line of this kind during a design phase.

Five test cases with varied heater power were applied to simulate the corresponding dynamic loads from the tested SRF modules operating at varied accelerating RF voltages after the installation of the helium transfer system. Actual value 1.287 of the tolerance factor is obtained from the heat load measurements of these five test cases. The corresponding rates of mass flow and pressure drops under varied operating conditions were measured concurrently. We reconfirm the feasibility and validity of our analytical approach with actual tolerance factor 1.287. Our calculated results are found to agree satisfactorily with the measured data after applying actual tolerance factor 1.287, which is less than safety factor 1.5 commonly adopted in cryogenic-related designs. The discrepancies of the pressure drops between the measured and calculated values for the LHe and CGHe transfer lines are an underestimate 0.11 kPa and an overestimate 0.09 kPa, respectively, which are much smaller than the measurement uncertainty.

Our model reveals also that roughly one sixth (17.0%) of the static heat load comes from the 200-m concentric flexible cryogenic transfer lines of corrugated type, one fifth (19.7%) from the horizontally oriented bayonet joints, and one-third (35.4%) from the short but non-LN_2_-shielded flexible lines for easy piping. The upstream rigid multi-channel lines as well as two cryogenic valve boxes, VB #1 and VB #2, account for 27.9% of the static heat load. This information provides one direction to upgrade or to optimize the present cryogenic transfer lines for a subsequent application. The design concept and the calculation approach of a pressure drop for a long helium transfer line developed herein is verified in the example of the 220-m flexible cryogenic transfer lines as built at NSRRC; we suggest that it is extensible to applications of varied cryogenic loads subject to similar operational constraints.

## Abbreviations

*A*: cross-sectional area of a pipe
Comp: compressor
CB: cold box
CGHe: cold gaseous helium
*D*_*h*_: hydraulic diameter of a pipe
*f*: friction factor
*F*_*B*_: tolerance factor
$$ \bar{g} $$
acceleration of gravity
$$ \bar{G} $$
mass flux
*h*: enthalpy
*K*_*L*_: loss coefficient, *K*_*L*_ = ∆*P*/(1/2*ρu* ^2^)
$$ K_{{\nu ,\max }} $$
maximum valve coefficient
$$ \ell $$
length of a pipe
LHe: liquid helium
$$ \dot{m} $$
mass flow rate
*M*: number of experimental data
ME: mean error
MPE: mean percentage error
MD: main Dewar
*P*_1_: upstream pressure of a valve
*q*: heat load
*R*: rangeability of a valve
Re: Reynolds number
SRF: superconducting radio frequency
SVB: switching valve box
S0: tested SRF module
S1: SRF module operated routinely in Taiwan Light Source
*T*_1_: upstream temperature of a valve
*u*: velocity of a fluid
VB: valve box
$$ \dot{V} $$
volumetric flow rate
*x*: vapor quality
*X*: flash ratio
*z*: direction along an inclined pipe

### Greek symbols

∆*P*: pressure drop
*ε*: surface roughness of a pipe
*θ*: inclined angle of a pipe
*μ*: dynamic viscosity of a fluid
*ρ*: density of a fluid
*Φ*: two-phase frictional multiplier term

### Superscripts

$$ \bar{L} $$
effective valve opening

### Subscripts

1*φ*: single-phase flow
2*φ*: two-phase flow
*calc*: calculation
*f*: frictional
g: gaseous phase
G: saturated gas of two-phase flow
L: saturated liquid of two-phase flow
*meas*: measurement
*s*: static
*T*: total
*val*: valve
*Ven*: Venturi-type flow meter
